# Development and characterization of an experimental model of diet-induced metabolic syndrome in rabbit

**DOI:** 10.1371/journal.pone.0178315

**Published:** 2017-05-23

**Authors:** Oscar Julián Arias-Mutis, Vannina G. Marrachelli, Amparo Ruiz-Saurí, Antonio Alberola, Jose Manuel Morales, Luis Such-Miquel, Daniel Monleon, Francisco J. Chorro, Luis Such, Manuel Zarzoso

**Affiliations:** 1 Health Research Institute (INCLIVA), Valencia, Spain; 2 Department of Physiology, Universitat de València, Valencia, Spain; 3 Department of Pathology, Universitat de València, Valencia, Spain; 4 UCIM, Universitat de València, Valencia, Spain; 5 Department of Physiotherapy, Universitat de València, Valencia, Spain; 6 Department of Cardiology, Clinic Hospital of Valencia, Valencia, Spain; 7 CIBERCV, Instituto de Salud Carlos III, Madrid, Spain; Universidad Miguel Hernandez de Elche, SPAIN

## Abstract

Metabolic syndrome (MetS) has become one of the main concerns for public health because of its link to cardiovascular disease. Murine models have been used to study the effect of MetS on the cardiovascular system, but they have limitations for studying cardiac electrophysiology. In contrast, the rabbit cardiac electrophysiology is similar to human, but a detailed characterization of the different components of MetS in this animal is still needed. Our objective was to develop and characterize a diet-induced experimental model of MetS that allows the study of cardiovascular remodeling and arrhythmogenesis. Male NZW rabbits were assigned to control (n = 15) or MetS group (n = 16), fed during 28 weeks with high-fat, high-sucrose diet. We measured weight, morphological characteristics, blood pressure, glycaemia, standard plasma biochemistry and the metabolomic profile at weeks 14 and 28. Liver histological changes were evaluated using hematoxylin-eosin staining. A mixed model ANOVA or unpaired t-test were used for statistical analysis (P<0.05). Weight, abdominal contour, body mass index, systolic, diastolic and mean arterial pressure increased in the MetS group at weeks 14 and 28. Glucose, triglycerides, LDL, GOT-AST, GOT/GPT, bilirubin and bile acid increased, whereas HDL decreased in the MetS group at weeks 14 and 28. We found a 40% increase in hepatocyte area and lipid vacuoles infiltration in the liver from MetS rabbits. Metabolomic analysis revealed differences in metabolites related to fatty acids, energetic metabolism and microbiota, compounds linked with cardiovascular disease. Administration of high-fat and high-sucrose diet during 28 weeks induced obesity, glucose intolerance, hypertension, non-alcoholic hepatic steatosis and metabolic alterations, thus reproducing the main clinical manifestations of the metabolic syndrome in humans. This experimental model should provide a valuable tool for studies into the mechanisms of cardiovascular problems related to MetS, with special relevance in the study of cardiovascular remodeling, arrhythmias and SCD.

## Introduction

Sedentary lifestyle and excessive caloric intake from processed food rich in fat and sugar have positioned obesity and the metabolic syndrome (MetS) as a real epidemic worldwide [1]. Even though there are several definitions of MetS, most of them describe it as a cluster of cardiovascular and metabolic alterations such as abdominal obesity, reduced HDL and elevated LDL cholesterol, elevated triglycerides, glucose intolerance and hypertension [2,3]. Diagnosis requires that any three out of these five criteria are present. MetS is associated with an increased risk of cardiovascular disease, non-alcoholic fatty liver disease and type 2 diabetes, leading to enormous financial burdens for healthcare systems [4]. Besides, the occurrence of life-threatening cardiac arrhythmias and sudden cardiac death (SCD) is quite high in patients with dyslipidemia, diabetes mellitus, hypertension or obesity. Indeed, epidemiological studies have shown that obese and diabetic patients have approximately twice and three times the risk, respectively, of SCD compared with age-matched controls [5–7].

Despite the increasing prevalence of MetS, the understanding of its pathophysiology and relationship with cardiovascular remodeling, cardiac arrhythmias and SCD is still very limited. Animal models are a powerful tool in understanding the mechanisms that underlie pathological processes such as MetS. In the case of cardiac remodeling and arrhythmogenesis, animal models, particularly rodent (rat and mouse) have provided valuable insights regarding the mechanisms involved in different pathophysiological abnormalities of the heart. However, in the context of studying the electrophysiological remodeling, small rodent models have many limitations. The murine ventricle displays a short triangular action potential (around 50 ms) and lacks the key repolarizing ion channels that are present in the human ventricle, namely, the fast and the slow delayed rectifier K^+^ currents (*I*_Kr_ and *I*_Ks_) [8]. On the other hand, in the rabbit ventricular myocardium, the action potential duration is approximately 200–300 ms, the morphology is spike and dome shaped, and the repolarization is mainly mediated by *I*_Kr_ and *I*_Ks_, which is similar to the human ventricle [9]. In addition, the rabbit model has been widely used for studying sustained arrhythmias and ventricular fibrillation [10,11], which is the main cause of SCD.

The establishment of an appropriate experimental model is needed to gain an understanding of the remodeling that takes place in the different organs and systems. To date, in the case of MetS, few diet-induced rabbit models using high-fat and high-sucrose diet have been used [12–16] and most importantly, a characterization of the different components of MetS has not been detailed. This is of great importance when relating a phenotype with organ remodeling. Thus, our main objective is to develop and characterize a diet-induced experimental model of MetS in rabbits that would later allow the study of cardiovascular remodeling and arrhythmogenesis.

## Material and methods

### Animals and diets

Animal care and the experimental protocols used in this study complied with EU directive 2010/63 on the protection of animals used for scientific purposes, and were approved by the Institutional Animal Care and Use Committee of the University of Valencia (2015/VSC/PEA/00049). Adult male New Zealand White (NZW) rabbits (n = 31) weighing 4.55±0.18 kg and 16–18 weeks old at the beginning of the experimental protocol were used in the present study. They were housed in a room with humidity (50±5%) and temperature (20±1.5^°^C) controlled conditions with a 12-h light cycle. After an acclimation of 3 weeks in which rabbits were fed 120 g of standard rabbit chow (V2333-000, Ssniff, Soest, Germany), animals were randomly assigned to a control (n = 15) or MetS group (n = 16). Control animals followed the same dietary regime, which has been shown to be appropriate for the maintenance of the adult rabbit (Carroll *et al*. 1996). Animals in the MetS group were fed *ad libitum* during 28 weeks with added high-fat (10% hydrogenated coconut oil, 5% lard; S9052-E020, Ssniff, Soest, Germany) and high-sucrose (15% dissolved in water) diet. Control diet contained 23.4% protein, 11.1% fat and 65.5% carbohydrates (2.7 kcal g^-1^). High-fat chow was composed mainly by 15.7% protein, 43.1% fat and 41.2% carbohydrates (3.7 kcal g^-1^), and the animals consumed 0.6 kcal mL^-1^ in the drinking solution. Food intake was registered daily, and assessed from the weight of the ingested chow and the volume of the consumed drinking solution. From these measurements, the weekly intake (kcal) was estimated.

### Morphological measurements

Body measurements were taken by means of measuring tapes and a weighing scale before administration of the experimental diet and at weeks 14 and 28. Body length, height, tibial length, abdominal circumference and abdominal circumference/body length ratio were determined. Bodyweight was measured in a weekly basis. Body mass index (BMI) was calculated as bodyweight (kg) [body length (m) × height (m)]^-1^ [17].

### Glycaemia and glucose tolerance test

Fasting glucose measurements were made before administration of the experimental diet and at weeks 14 and 28 with a glucose meter (Contour Next, Bayer, Leverkusen, Germany). For the evaluation of glucose metabolism, intravenous glucose tolerance test (IVGTT) was performed as previously described [11]. Briefly, rabbits were fasted during 7 hours and then the experiment started between 14 and 15 p.m. After auricular vein cannulation, a bolus of a 60% glucose solution (0.6 g kg^-1^) was administered i.v. through the marginal ear vein and blood samples were taken before and at different time points after injection (15, 30, 60, 90, 120 and 180 minutes). Blood glucose was measured with a glucose meter (Contour Next, Bayer, Leverkusen, Germany). The area under the curve (AUC) was calculated by multiplying the cumulative mean height of glucose (mg dL^-1^) by the time (hours) [18].

### Blood pressure

Rabbits were restrained in a plastic holder and, after topical local anaesthetic application (EMLA, Astra AstraZeneca, Madrid, Spain), the central auricular artery was cannulated (Introcan 18G, Braun, Melsungen, Germany). Then restraints were loosened and the rabbit was allowed to stay quietly for 20–30 min. Blood pressure was recorded directly from the arterial catheter, using a pressure transducer positioned at the heart level. The signal from the transducer (Model 60–3003, Harvard Apparatus, Holliston, MA) was amplified, sent to a Power Lab unit (Power Lab 2/26, AD Instruments, Oxford, UK), and then registered with Labchart (ver. 6, AD Instruments, Oxford, UK) with a sampling frequency of 1 KHz. Data were analyzed using custom-made software and the last 5 minutes of a 20 minutes recording were processed to calculate systolic, diastolic, and mean arterial pressure (MAP). Determinations were made at weeks 14 and 28.

### Plasma measurements

Blood samples were taken from the auricular vein at week 14 and 28. After 7 hours fasting, samples were collected in EDTA tubes (BD Vacutainer, Plymouth, UK), stored on ice and centrifuged (1500 g, 15 min, 4^°^C). Then, plasma was obtained and stored in a -80^°^C refrigerator. Plasma triglycerides, total cholesterol, HDL, LDL, transaminases, gamma-glutamyl transpeptidase (GGT), bile acid, bilirubin, creatinine, urea, total protein, albumin, glucose and creatine phosphokinase (CPK) were determined using standard enzymatic procedures by an external laboratory (Immunovet, Barcelona, Spain).

### Histology

Liver tissue was obtained from 5 animals (control n = 3, MetS n = 2). Following premedication with ketamine (35 mg kg^-1^) and heparine (2500 IU), rabbits were euthanized with an overdose of sodium pentobarbitone (100 mg kg^-1^). Then, the liver was carefully removed and immersed in a 4% formaldehyde solution, embedded in paraffin, serially sectioned in 5 μm, mounted in adhesive slides and stained with hematoxylin and eosin. We performed a morphometric study in 5 microphotographs for each animal in the area of the central veins, by means of a DMD108 microscope (Leica Mircrosystems, Wetzlar, Germany) and a 40x objective. Image-Pro Plus 7.0 was used for image analysis and the following parameters were quantified: hepatocyte number, area, and number of hepatocytes with lipid vacuole content.

### Nuclear magnetic resonance (NMR) Spectroscopy

Plasma samples (450 μL) from both control and MetS group were mixed with 45 μL of TSP solved in D_2_O and placed in a 5-mm NMR tube. Final concentration of TSP in each sample was 5 mM. 1H NMR spectra were recorded using a Bruker Avance DRX 600 spectrometer (Bruker GmbH, Rheinstetten, Germany). Samples were measured at 37°C and a single-pulse pre-saturation experiment was acquired in all samples. The water signal was saturated with weak irradiation during the recycle delay. All spectra were processed using MestReNova 8.1 (Mestrelab Research S.L., Spain). Data were Fourier transformed after the free induction decay was multiplied by a 0.3 Hz exponential line-broadening function. The spectra were manually phase corrected and baseline adjusted, referenced to TSP and normalized to TSP to eliminate differences in metabolite total concentration. The Chenomx NMR Suite Profiler (Chenomx NMR Suite 8.1, Alberta, Canada) was used to identify signals belonging to selected metabolites by fitting compound signatures from the provided NMR spectral library. In total, 59 metabolites were identified and the concentration of each sample was calculated by determining the heights of the compound signatures that best fit the sample spectra with the effective concentration of the internal TSP standard 5 mM. In addition, two-dimensional NMR methods including homonuclear correlation spectroscopy (TOCSY) and heteronuclear single quantum correlation spectroscopy (HSQC) were used to confirm the assessment of metabolites.

### Statistical analysis

Values are reported as mean ± SD unless stated otherwise. Shapiro-Wilk test was performed on all variables to assess the normality of the distributions. Unpaired t-test and a mixed model ANOVA with two factors, one within-subjects (time) and one between-subject (group), were used for statistical analysis when appropriate. Bonferroni correction was applied for pairwise comparisons. Differences were considered significant at a two-tailed alpha level of P<0.05.

## Results

### Morphological parameters and energy intake

**Fig 1:**
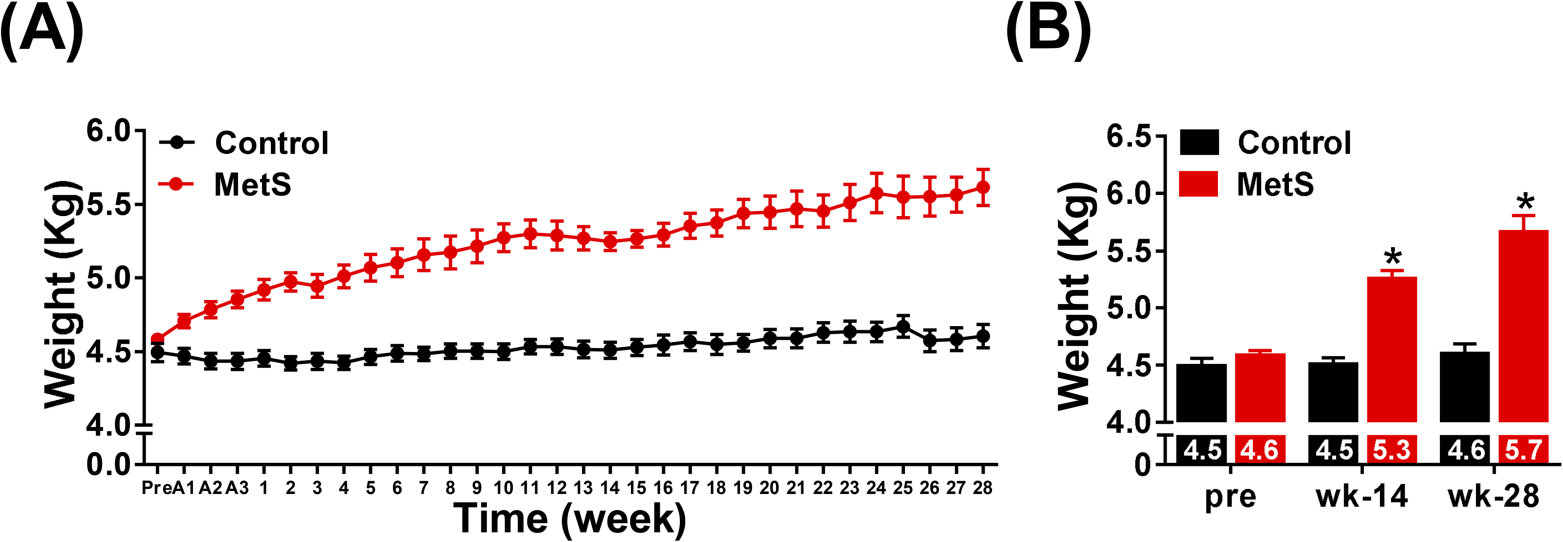
Weight evolution during the experimental period. Weekly weight measurement is displayed in panel (A), while comparison of weight increase at weeks 14 and 28 are shown in panel (B). Control (n = 12), MetS (n = 13), A*n* = acclimation week, *p<0.05 *vs*. control, error bars display SEM.

There was a progressive weight gain in the MetS group that started to be evident as early as the first week of adaptation to the diet (Fig 1A). At week 14, animals in MetS group showed a 17.8% increase in body weight compared with controls (5.26±0.2 *vs*. 4.5±0.2 kg, p<0.05, Fig 1B) and, by the end of week 28, this increase reached 23.9% (5.66±0.4 *vs*. 4.6±0.3 kg, p<0.05, Fig 1B). Regarding morphological parameters, since adult rabbits were used in the study, we did not find any difference between the two groups in body length, height or tibial length, or when comparisons were made within groups (Table 1). On the other hand, abdominal circumference, abdominal circumference/length ratio and BMI increased in MetS animals when compared to controls (Table 1). No difference was found within control group between pre-diet and weeks 14 and 28, whereas these parameters increased in MetS animals (Table 1).

**Table 1:** Morphological characteristics.

	Pre-diet		Week 14		Week 28	
	Control	MetS	Control	MetS	Control	MetS
**Length (cm)**	53.6±1.9	53.4±2.1	53.5±1.8	53.9±1.7	53.8±1.1	54.4±1.9
**Height (cm)**	25.9±0.8	25.7±1.4	26.2±0.5	26.2±0.5	26.1±1.3	25.9±1.4
**Abd. circumference (cm)**	41.5±2.1	41.6±2.1	41.1±2.1	47.2±2.1*^#^	40.0±1.2	49.0±3.1*^#^^$^
**Tibial length (cm)**	16.5±0.6	16.5±0.6	16.8±0.6	16.7±0.3	16.8±0.3	16.8±0.5
**Abd. circ./ length (cm)**	0.77±0.04	0.77±0.04	0.75±0.08	0.87±0.05*^#^	0.72±0.1	0.89±0.01*^#^
**BMI (Kg/m**^**2**^**)**	32.4±1.7	32.8±2.5	32.3±1.2	37.6±1.9*^#^	32.9±2.5	40.2±4.2*^#^^$^

Control (n = 11) and MetS (n = 13).

*p<0.05 *vs*. control

^#^p<0.05 *vs*. pre

^$^p<0.05 *vs*. week 14.

**Fig 2:**
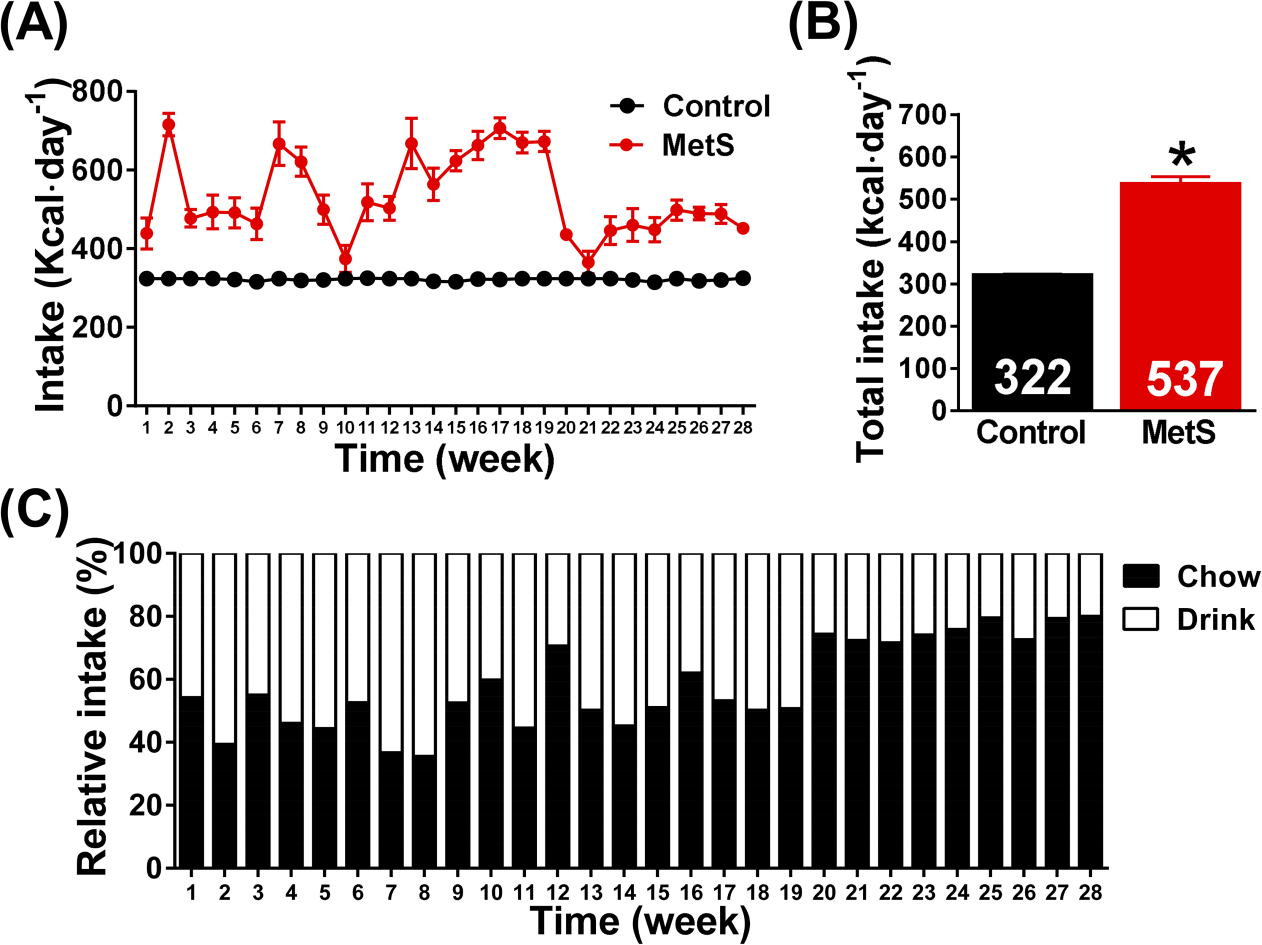
Energy intake in the experimental groups. Panel (A) illustrates the evolution of weekly intake during the 28 weeks of the experimental period. In panel (B), mean caloric intake is displayed. The relative intake in percentage of kcal from high-fat chow and the drinking solution can be observed in panel (C). Control (n = 6), MetS (n = 8), *p<0.05 *vs*. control, error bars display SEM.

Daily intake fluctuated in MetS animals during the 28 weeks of diet administration (ranging between 716 and 374 kcal day^-1^), while intake in control group remained constant since animals normally consumed the 120 g of chow that were provided for maintenance (Fig 2A). When the mean value of the 28 weeks was computed, we obtained that MetS rabbits ingested 66.7% more kcal than controls (537±41 *vs*. 322±8 kcal day^-1^, p<0.05, Fig 2B). In panel C the relative intake of chow and drink is displayed, and we could observe an apparent switch of the balance between the amount of kcal from drinking solution towards an increase in the caloric intake from chow around the week 20.

### Effects of high-fat, high-sucrose diet on glucose metabolism

**Fig 3:**
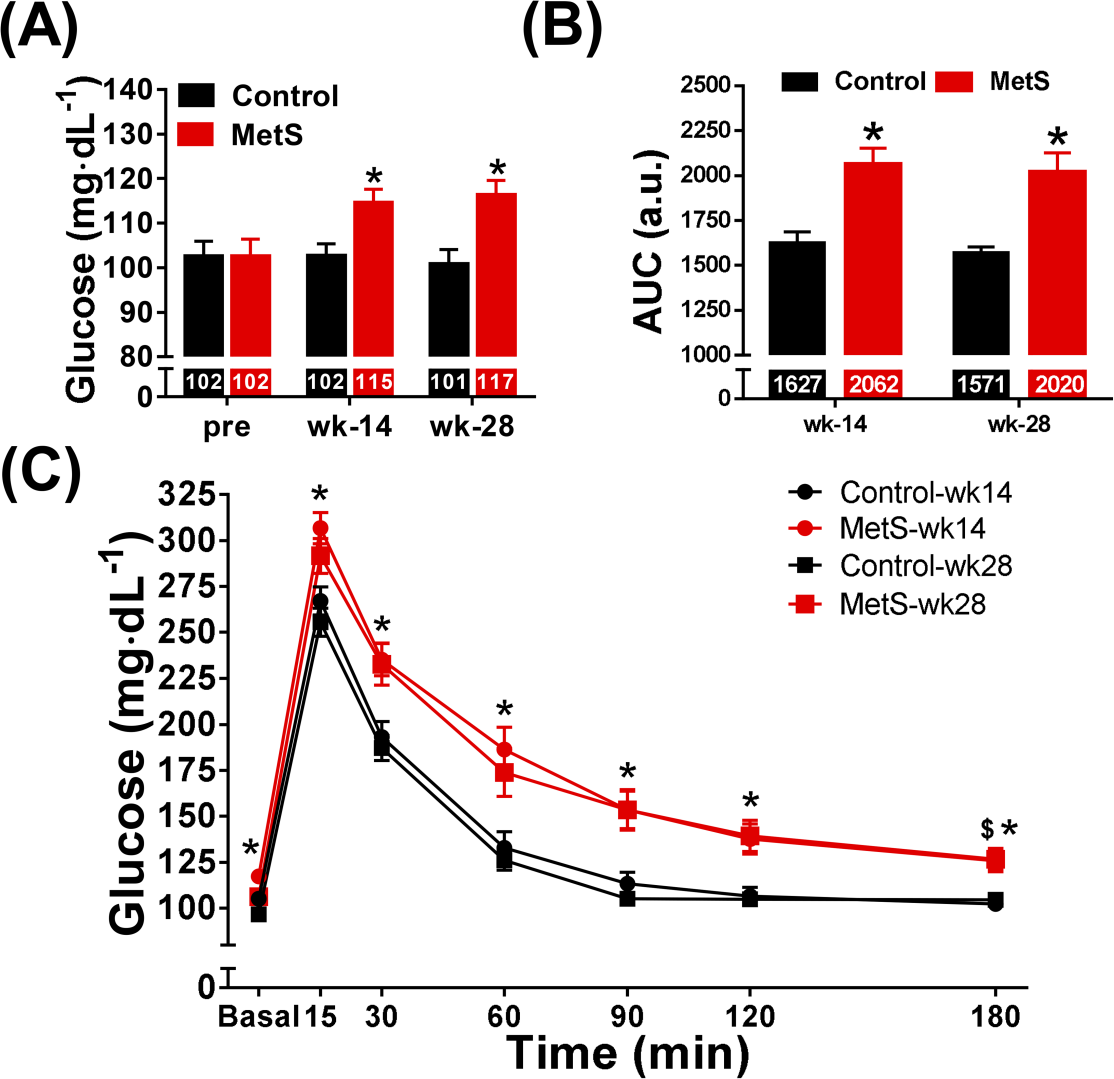
Blood glucose regulation. Blood glucose measurements were taken after fasting before diet and at 14 and 28 weeks of high-fat, high-sucrose administration (A). The results of the IVGTT at weeks 14 and 28 are shown in panel (C) and the quantification of the area under the curve (AUC) is depicted in panel (B). Control (n = 12), MetS (n = 13), *p<0.05 *vs*. control, ^$^p<0.05 vs basal, error bars display SEM.

Fasting glucose levels were similar between groups before diet administration (102±10 *vs*. 102±13 mg dL^-1^). High-fat, high-sucrose diet increased fasting glucose levels in MetS rabbits at weeks 14 (115±10 *vs*. 102±7 mg dL^-1^, p<0.05) and 28 (117±11 *vs*. 101±10 mg dL^-1^, p<0.05, Fig 3A).

Additionally, an IVGTT was performed. After fasting, baseline plasma glucose levels were increased in MetS rabbits (117±11 *vs*. 105±8 mg dL^-1^ at week 14, 110±12 *vs*. 96±6 mg dL^-1^ at week 28, p<0.05, Fig 3C). MetS rabbits showed higher blood glucose values and a slower rate of glucose clearance from the blood than control animals, at all the time points studied after 15 min of intravenous glucose injection, at both weeks 14 and 28 (Fig 3C). Animals in the control group reached plasma glucose levels similar to baseline after 180 minutes (102±12 *vs*. 105±8 mg dL^-1^ at week 14, 104±9 *vs*. 96±6 mg dL^-1^ at week 28, Fig 3C). On the other hand, this parameter remained increased after 180 minutes in MetS rabbits at weeks 14 (126±21 *vs*. 117±11 mg dL^-1^, p<0.05 *vs*. basal) and 28 (127±21 *vs*. 106±12 mg dL^-1^, p<0.05 *vs*. basal, Fig 3C). We did not find differences in plasma glucose levels in response to IVGTT between week 14 and 28 when comparisons were made within groups. The AUC calculated from the IVGTT increased in the MetS group at weeks 14 (2062±330 *vs*. 1627±184 a.u., p<0.05) and 28 (2019±381 *vs*. 1570±99 a.u., p<0.05, Fig 3B) when compared with the control group.

### MetS-induced hypertension

**Fig 4:**
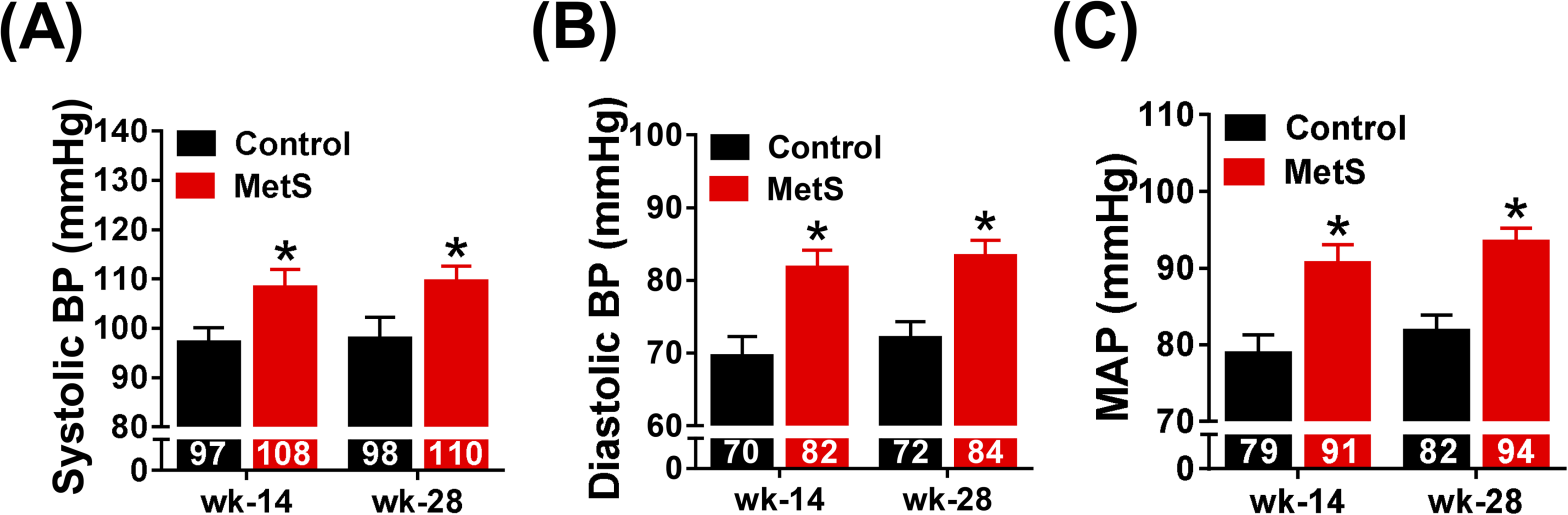
Modifications in blood pressure. Panels (A) and (B) show systolic and diastolic blood pressure measurements at week 14 and 28 in both experimental groups. Mean arterial pressure (MAP) is presented in panel (C). Control (n = 10), MetS (n = 11), *p<0.05 *vs*. control, error bars display SEM.

Blood pressure measurements were made through the auricular artery in conscious animals and revealed that MetS rabbits developed mild hypertension. Systolic and diastolic blood pressure increased at weeks 14 (systolic 108.8±13.2 *vs*. 96.7±9.3 mmHg, diastolic 82.2±8.1 *vs*. 69.8±8.8 mmHg, p<0.05) and 28 (systolic 110.4±9.3 *vs*. 98.3±9.8 mmHg, diastolic 84.3±6.5 *vs*. 72.5±4.7 mmHg, p<0.05) in MetS rabbits with respect to controls (Fig 4A and 4B), but no difference was found within groups between weeks 14 and 28. Consequently, MAP showed a 15.2 and 14.6% increase at weeks 14 (92.0±8.4 *vs*. 78.6±8.1 mmHg, p<0.05) and 28 (94.4±5.3 *vs*. 82.1±3.2 mmHg, p<0.05), respectively, in the MetS group (Fig 4C).

### Changes in plasma metabolites

**Fig 5:**
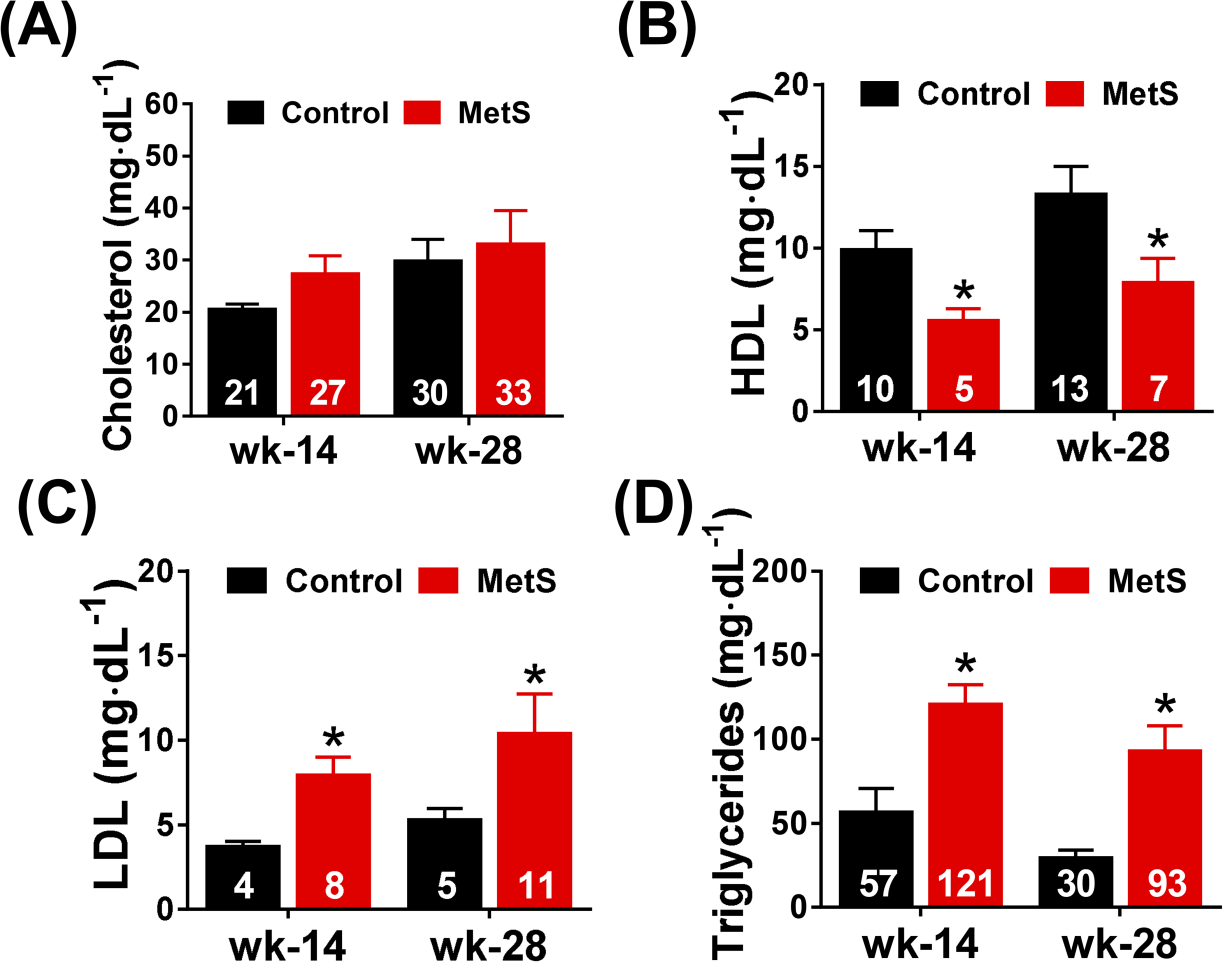
Lipid profile. Plasma measurements of total cholesterol (A), HDL (B), LDL (C) and tryglicerides (D) at weeks 14 and 28. Control (n = 11), MetS (n = 13), *p<0.05 *vs*. control, error bars display SEM.

We further examined if the metabolic disorders of the MetS modified the lipid profile. Indeed, we found an increase in both LDL and triglycerides and a decrease in HDL at weeks 14 and 28 (Fig 5B–5D). No difference was found within groups comparing between week 14 and 28. Total cholesterol levels were not different between control and MetS rabbits, nor did we observe differences within groups over time (Fig 5A). Total protein increased in MetS rabbits when compared to controls at weeks 14 and 28, but this increase was not due an increase in albumin, which remained unchanged in both groups (S1 Table). CPK increased in MetS animals with respect to controls at week 28, but we did not find changes at week 14 probably due to the high variability in the measurements (S1 Table). Even though there was no difference in creatinine between or within groups, urea decreased in the MetS groups at weeks 14 and 28 compared with the control group (S1 Table).

**Fig 6:**
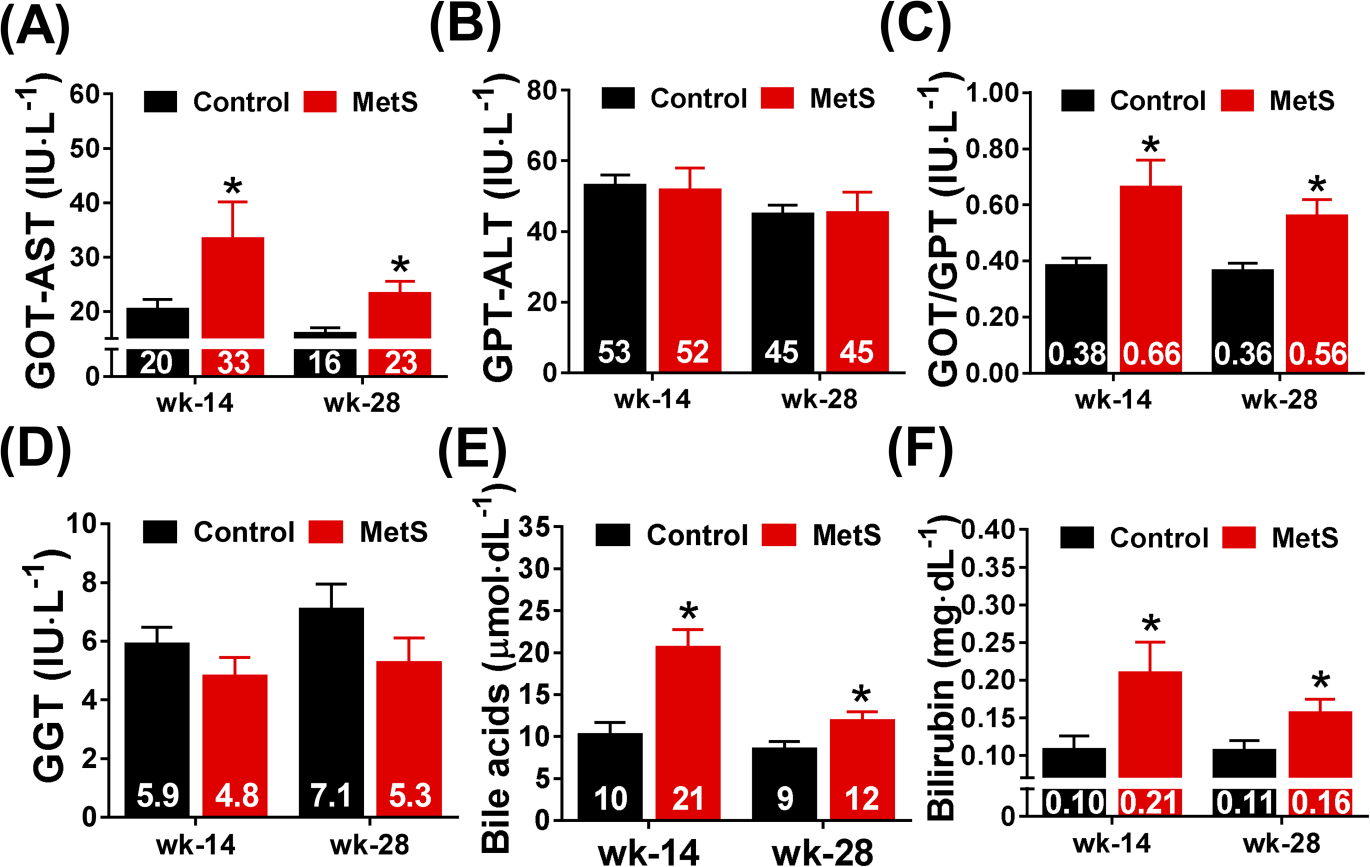
Liver damage markers at week 14 and 28. Plasma measurements of transaminases (GOT-AST, GPT-ALT and GOT/GPT ratio) are depicted in panels (A-C). Other liver function markers such as gamma-glutamyl transferase (GGT), bile acid and bilirubin are shown in panels (D-F). Control (n = 11), MetS (n = 13), *p<0.05 *vs*. control, error bars display SEM.

Finally, we analyzed the effect of high-fat, high-sucrose diet on liver damage markers. GOT-AST and GOT/GPT ratio increased in animals from MetS group at weeks 14 and 28 (Fig 6A and 6C), whereas we did not find changes in GPT-ALT and GGT (Fig 6B and 6D). There was no difference in these parameters when comparisons were made within groups between weeks 14 and 28. On the other hand, both bile acids and bilirubin increased in MetS rabbits at week 14 and 28 (Fig 6E and 6F), but in a similar way to what happened with transaminases, we did not find any difference within groups over time.

### Histological examination

**Fig 7:**
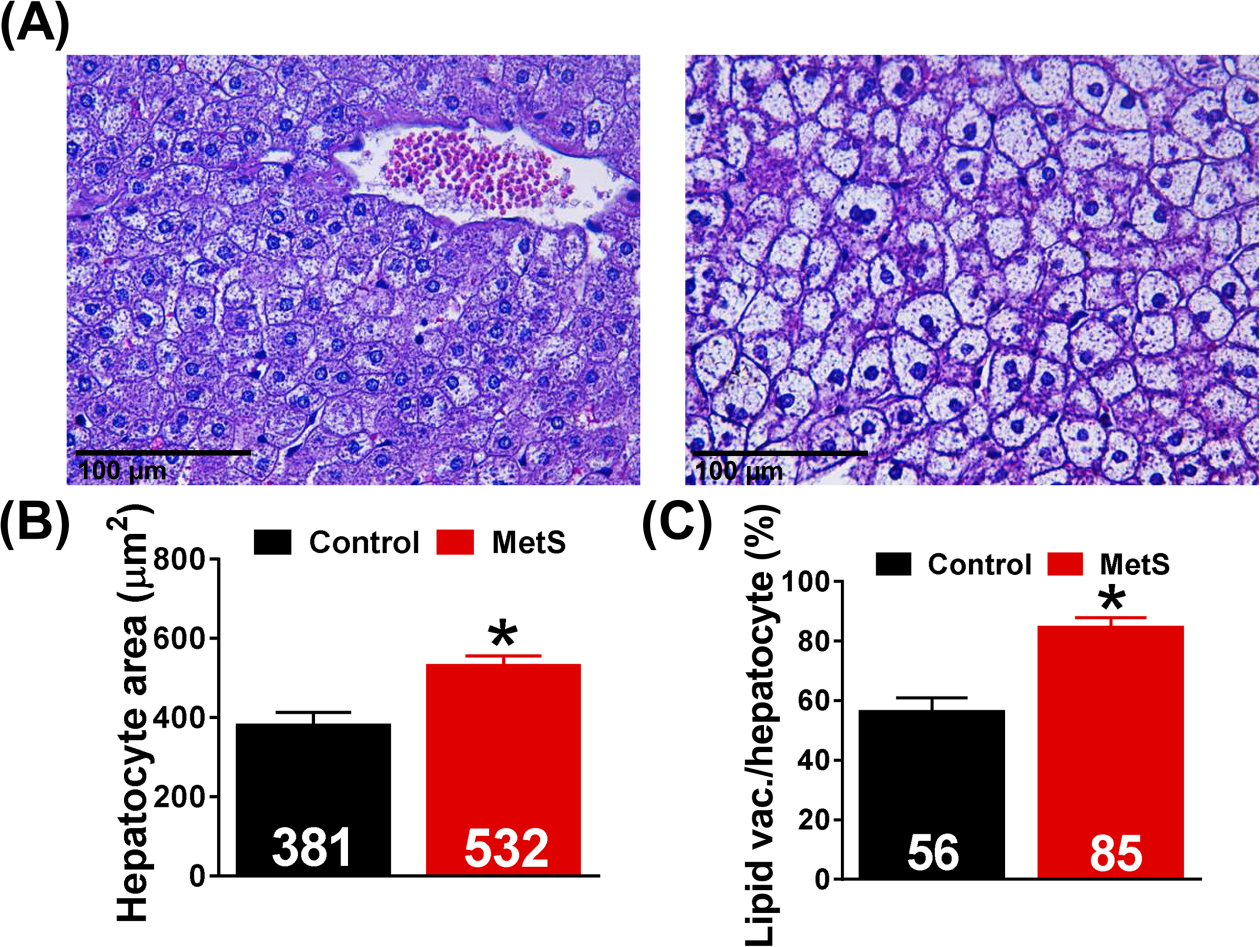
Liver histological study. Representative microphotographs (central veins area) after hematoxylin-eosin staining are shown in panel (A) for control (left) and MetS (right). Quantification of hepatocyte area and lipid vacuole/hepatocyte ratio is displayed in panels (B) and (C), respectively. Control (N = 3, n = 15), MetS (N = 2, n = 10), *p<0.05 *vs*. control, error bars display SEM.

We found a 40% increase in hepatocyte area in the liver from MetS rabbits (532±24 *vs*. 381±32 μm^2^, p<0.01, Fig 7B), showing an enlargement of hepatic cells in this group. Most importantly, the percentage of hepatocytes in which lipid vacuoles were present increased in rabbits from MetS group (84.7±3.3 *vs*. 56.4±4.7%, p<0.001, Fig 7C), pointing towards the development of hepatic steatosis.

### Metabolomic analysis

The metabolomic profile revealed differences in metabolites related to fatty acids. Indeed, FA(-CH_2_-)n, FA-β-CH_2_, FA-CH_2_C = C, FA-α-CH_2_ and FA-CH = CH increased in rabbits from MetS group at week 14 and 28, but no difference was found within groups comparing week 14 and 28 (Table 2). Other key metabolites for energetic metabolism such as pyruvate and lactate, and diet-related metabolites (sucrose and mannose) were increased in MetS rabbits (Table 2). We also found an increase in amino acids like alanine and glycine at week 14 in the MetS group, while at week 28 differences were reported in alanine and threonine (Table 2). Acetoin, a metabolite which has been linked with gut microbiota, increased in MetS animals at weeks 14 and 28 (Table 2). Finally, other compounds such as allantoin, related to oxidative stress, decreased in MetS rabbits at weeks 14 and 28 when compared to controls (Table 2). We did not find any difference between groups in the rest of metabolites that were identified (S2–S4 Tables).

**Table 2:** Modifications of metabolites as shown by metabolomic analysis.

	Week 14		Week 28	
	Control	MetS	Control	MetS
**Acetoin**	1.1±0.3	1.6±0.5*	0.9±0.1	1.3±0.5*
**Alanine**	1.9±0.3	2.6±0.5*	1.7±0.3	2.2±0.5*
**Allantoin**	0.12±0.04	0.10±0.02*	0.15±0.02	0.11±0.04*
**FA(-CH**_**2**_**-)n**	11.9±4.4	18.2±7.2*	9.5±1.1	16.1±7.5*
**FA-β-CH**_**2**_	7.2±0.8	7.9±1.0	6.9±0.4	7.9±0.9*
**FA-CH**_**2**_**C = C**	6.0±0.7	6.6±0.8*	5.7±0.3	6.4±0.7*
**FA-α-CH**_**2**_	3.1±0.6	3.8±0.6*	2.8±0.2	3.6±0.6*
**FA-CH = CH**	1.2±0.6	1.5±0.7	0.9±0.1	1.4±0.5*
**Glycine**	0.7±0.1	1.0±0.2*	0.7±0.1	0.8±0.1
**Lactate**	6.6±1.5	9.8±4.1^#^	4.9±1.2	8.3±4.0*
**Mannose**	0.8±0.3	1.2±0.6	0.6±0.2	0.9±0.3*
**Pyruvate**	0.33±0.04	0.45±0.1*	0.30±0.05	0.43±0.07*
**Sucrose**	0.64±0.17	0.91±0.37^#^	0.72±0.16	0.94±0.30*
**Threonine**	1.8±0.4	2.1±0.3	1.5±0.2	2.1±0.4*

Control (n = 10) and MetS (n = 11).

*p<0.05 *vs*. control

^#^p = 0.06 *vs*. control.

## Discussion

The aim of this study was to develop a diet-induced experimental model of MetS in NZW rabbits and perform a detailed characterization of the main components that define human MetS. Our results showed that administration of high-fat and high-sucrose diet during 28 weeks induced 1) central obesity, 2) a state of prediabetes characterized by impaired fasting glucose and glucose intolerance, 3) mild hypertension, 4) alterations in the lipid profile revealed by an increase in triglycerides and LDL, a decrease of HDL and no changes in total cholesterol, 5) liver damage, as shown by the increase in GOT-AST, GOT/GPT ratio, bile acids and bilirubin 6) liver steatosis and 7) modifications in the metabolism of lipids, proteins, carbohydrates and gut microbiota which have been linked with cardiovascular disease. Most importantly, we have developed a relevant and inexpensive animal model of diet-induced MetS which mimics the main changes that occur in humans.

Animal models are a powerful tool in understanding the mechanisms that underlie pathological processes such as MetS. To date, diet-induced spontaneous and transgenic rodent models have been widely used to study the effect of MetS in different organs and systems. Although rodent MetS models can exhibit some of the components of this complex pathology, mice and rats are deficient in cholesteryl ester transfer protein (CETP) and thus resistant to the development of atherosclerosis and coronary heart disease, due to important differences in lipid metabolism with humans [19]. In addition, several rodent models do not develop important characteristics that are associated with MetS such as hypertension, hyperglycemia and hyperinsulinemia [20]. Furthermore, obesity in some transgenic models is not dependent solely on dietary factors and some strains can become obese with normal or even decreased food intake [21]. Larger canine and swine animal models have also been used, but some argue about the utility of the former given that dogs, as it happens with rodents, do not develop atherosclerosis or hyperglycemia [22]. Swine models, due to the similarity with human anatomy and physiology, may have significant predictive power for elucidating the pathophysiology of MetS [23], but their maintenance is necessarily very labor intensive and costly. The rabbit, on the other hand, is flexible enough for many different types of studies while avoiding some of the drawbacks of large animal models and most importantly, like humans, the rabbit has abundant CETP in plasma, which acts as an important regulator of reverse cholesterol transport, and their lipoprotein profile is LDL-rich [14]. Rabbits, as herbivores, are very sensitive to dietary fat, therefore, they can rapidly develop hyperlipidemia [24]. In addition, it has been shown that rabbits fed with a high-fat diet have similar hemodynamic and neurohumoral changes as obese humans [21,11].

In the present study the diet was well tolerated and rabbits increased progressively in weight until the end of the experimental protocol. We observed a 24 and 22% increase in bodyweight and BMI, respectively, at week 28, showing that rabbits were obese but they did not reach a degree of severe obesity. This is a similar increase in weight obtained in previous studies with younger male rabbits [11] fed with high-fat diet, but far from the 45% increase obtained by Carroll *et al*. [21] in adult female rabbits. On the other hand, no increase in bodyweight was reported by others using high-fat and high-sucrose/fructose diet [12,15,16,18], but the amount of sucrose used for feeding, ranging from 30 to 40%, was not well tolerated by rabbits [12]. In our study, the use of 15% sucrose in water seemed to be better tolerated than higher proportions in the chow, and the increase in bodyweight was evident. The development of obesity is an important factor to take into account, since there is a close correlation between central obesity and the risk factors that define MetS [2]. Thus, this model shows similarities with human obesity, in which body weight gain comes mainly from an increase in abdominal fat mass, as suggested by the increase in abdominal circumference and abdominal circumference/length ratio. We also observed unhealthy coating conditions in rabbits in MetS group.

Excess weight gain and obesity are associated with hemodynamic alterations such as increased blood volume, preload, afterload and commonly hypertension. In this line, we observed an increase in systolic, diastolic and mean blood pressure at week 14 that was maintained at week 28. Previous studies using a high-fat and high sucrose diet administration during 36 weeks resulted in no change in blood pressure despite reporting a 22% increase in bodyweight [13], but the use of a different rabbit breed and anesthesia during the measurements could explain these differences.

Mild hyperglycemia was observed in MetS rabbits as early as week 14, as depicted by differences in fasting blood glucose. The rise on fasting glycemia level measured at week 14 was very modest and reached a plateau, since we did not observe a further increase at week 28. Our values are slightly below from those reported in the literature administering 30–40% fructose or glucose in the chow [12,18]. Of note, some studies found no difference in fasting glucose after high fat and high sucrose diet administration, but using different rabbit breeds [13,16]. Defects in glucose regulation were also present as evidenced by the results of the IVGTT. Indeed, the AUC and blood glucose levels after 180 minutes increased in the MetS group, which reflected the inability to recover baseline glucose levels. The precise mechanism underlying these abnormalities is not completely known, but insulin resistance in MetS has been attributed to the increase in adipose tissue and overabundance of circulating fatty acids and its accumulation [25,26]. On the other hand, diagnosis of type II diabetes in rabbits based on blood glucose has not been established, but we could conclude that rabbits in MetS group developed a state of pre-diabetes with impaired fasting glucose and glucose intolerance. Even though rabbits did not develop type 2 diabetes, the model could be useful to study the condition that precedes the clinical manifestation of the pathology, thus allowing the identification of preclinical markers that might allow the detection of patients at risk.

In our model, dyslipidemia appeared as early as week 14 and was maintained in week 28. Alterations in plasma lipid profile were characterized by an increase in triglycerides and LDL, a decrease of HDL and no changes in total cholesterol. This is similar to the criteria that have been established in humans for the diagnosis of MetS [2]. In addition, we also found a decrease in urea levels in plasma that could be explained by the reduced amount of protein present in the chow of MetS rabbits (15.7 *vs*. 23.4%). Conversely, total protein was increased in MetS rabbits at weeks 14 and 28 without changes in albumin, so this increase was probably due to an increase in globulins, some of which have been recently linked with the development of type 2 diabetes [27] and have been proposed as biomarkers for the development of liver fibrosis in non-alcoholic fatty liver disease [28].

Plasma analysis also enabled us to find signs of liver abnormalities as shown by the increase in GOT-AST, GOT/GPT ratio, bile acids and bilirubin at weeks 14 and 28. Modifications of liver damage markers are, when the pathology is not related with alcoholic steatosis, nonspecific and include an increase in GOT-AST, GPT-ALT, GGT and bilirubin. Our results are therefore compatible with the development of hepatic steatosis. When we examined liver histology after staining with hematoxylin-eosin and performing a morphometric study, an increase in hepatocyte area and in percentage of lipid vacuole infiltration was observed. These changes are also consistent with the development of hepatic steatosis which, despite not being included in the diagnosis criteria of MetS, often accompanies this cluster of metabolic abnormalities [29] and could lead to the development of steatohepatitis, fibrosis and cirrhosis. Similar results have been reported in Watanabe heritable hyperlipidemic rabbits after 16 weeks of high-fat (10%) and high-fructose (30%) feeding [16].

Nuclear magnetic resonance spectroscopy can be used to generate a “molecular fingerprint” of a serum sample to identify molecular signatures associated with MetS. There are few experimental studies that have examined modifications of plasma metabolome in response to diet interventions and, to our knowledge, this is the first characterization of the metabolomics changes in a diet-induced experimental model of MetS in rabbit. Metabolomics analysis enabled us to identify differences between groups at week 14, which in most cases where maintained in week 28, in metabolites involved in the metabolism of lipids, proteins, carbohydrates and microbiota.

Increases in plasma levels of amino acids have been identified as markers of obesity and insulin resistance. In this line, we found and increase in alanine and threonine, which have been reported to be augmented in type II diabetes and obesity [30,31] and has been proposed as a clinical predictor for the development of hyperglycaemia and type II diabetes [32]. In addition, we also found modifications in the metabolism of carbohydrates, as shown by the increase in the concentration of metabolites such as mannose, sucrose and lactate, which have also been associated with type II diabetes [33,34]. Defects in the mitochondrial respiratory chain and mitochondrial dysfunction as a significant factor in insulin resistance induced by fatty acids and type 2 diabetes is one of the critical areas in metabolic disorders, which is growing in importance [35]. We found an increase in pyruvate levels in plasma, which has been reported in patients with type 2 diabetes [36]. Furthermore, hyperglycemia might also boost the rate of glucose oxidation towards the excessive formation of pyruvate, indicating that the metabolic adaptations are directed to glycolytic pathways in the progression towards type 2 diabetes and insulin resistance [35].

In our study, high-fat and high-sucrose diet increased the spectral intensity of fatty acids moiety, FA(-CH_2_-)n, FA-β-CH_2_, FA-CH_2_C = C, FA-α-CH_2_ and FA-CH = CH, which in turn reflects total circulating fatty acids. Diets with a high fat content result in a number of metabolic perturbations including dyslipidaemia by altering the proportion of VLDL/LDL and HDL. In addition, fatty acids are independent predictors of progression to diabetes and impair insulin actions via mechanisms including the Randle cycle, accumulation of intracellular lipid derivatives (eg, diacylglycerol and ceramides), oxidative stress, inflammation, and mitochondrial dysfunction [37]. Many studies suggest that an increase in circulating free fatty acids as one of the main causes for this dyslipidemia [38,39].

The dysregulation of gut microbiota has been related to several metabolic diseases such as nonalcoholic fatty liver disease, nonalcoholic steatohepatitis, diabetes, insulin resistance and obesity [40]. It has been shown that high-fat diet induces metabolic changes and, among them, bile acids alterations that in turn can produce a remodeling of gut microbiota [41]. Even though we did not study bile acids composition, we did find an increase in bile acids in plasma as well as an increase in acetoin, a metabolite that can be produced in pyruvate metabolism or by gut microbiota [42–45].

The following limitations of our study should be considered: 1) Regarding central obesity, we measured abdominal circumference and BMI, but the determination of body fat distribution using magnetic resonance imaging would have been more adequate. 2) Animals in MetS group developed glucose intolerance as shown by the increase in fasting glucose and the AUC in the GTT, but insulin level was not determined so we cannot establish if the cause of glucose intolerance was insulin resistance or decreased insulin production. 3) Hematoxylin and eosin staining was used for liver histological evaluation and identification of lipids was made by negative vision. The use of Oil red-O would have provided a more accurate staining of lipid content. 4) We did not perform a detailed characterization of heart structural, functional or electrical remodeling with ECG or echocardiographic measurements, which should be addressed in future studies. Of note, the overall effects on metabolic profile were very similar between 14 and 28 weeks without an evident worsening, which could suggest that animals were facing an adaptation process. Even though we do not have actual data to explain this process, we could speculate with the combination of the following factors: 1) The use of young adult rabbits, since metabolic syndrome often occurs in middle-aged adults or older [46,47]. 2) The decrease in the intake from the drinking solution during the last third of the 28-week period (Fig 2C), which produced a decrease in sucrose ingestion and could be related with the stabilization of fasting glycaemia. The rest of measurements made in plasma samples followed the same trend. 3) Weight gain was evident in the second half of the study, but not as marked as the first half, in line with the decrease of intake (Fig 2A), which could also explain the stabilization of blood pressure since, as it is well-known, the relationship between hypertension and BMI is nearly linear [48].

Animal models are a powerful tool in understanding the mechanisms that underlie pathological processes such as MetS. Even though there are several animal models of MetS, it is difficult to choose a single model that appropriately represents the human condition and yet is suitable for cardiovascular studies. With this objective, we have described a new animal model of dietary-induced MetS that exhibits many of the characteristics of the pathology in humans. Furthermore, to our knowledge, the rabbit has not been previously used for the study of the different components of MetS and this is the first detailed characterization using a diet high in saturated fat and sucrose. Of note, the use of a diet-induced model is important since diet affects whole-body metabolism and regulation through effects on hormones, glucose metabolism, lipid metabolism pathways and its effects in the different organs, closely emulating what happens in human MetS.

In conclusion, we developed a relevant model of diet-induced MetS characterized by central obesity, hypertension, pre-diabetes and dyslipidemia with low HDL, high LDL and an increase of TG levels, thus reproducing the main clinical manifestations of the metabolic syndrome in humans. We also identified important changes in the metabolome directed towards the development of insulin resistance and type 2 diabetes as well as changes in liver morphology and function, which point to the development of hepatic steatosis. This experimental model should provide a valuable tool for future studies into the mechanisms of cardiovascular, hormonal or metabolic problems related to MetS, with special relevance in the study of cardiovascular remodeling, arrhythmias and SCD.

## Supporting information

S1 Table Plasma biochemistry. (PDF)

S2 Table Metabolomic analysis of fatty acids and related compounds. (PDF)

S3 Table Metabolomic analysis of amino acids. (PDF)

S4 Table Metabolomic analysis of other metabolites. (PDF)
