# Supplementary material for: Development and characterization of an experimental model of diet-induced metabolic syndrome in rabbit
Source: PLoS One. 2017 May 23;12(5):e0178315. doi: 10.1371/journal.pone.0178315 (PMC5441642; doi:10.1371/journal.pone.0178315)
Supplement: S1 Table — (PDF) [file pone.0178315.s001.pdf]

	Week 14		Week 28	
	**Control**	**MetS**	**Control**	**MetS**
**Albumin (g/dL)**	4.1±0.2	4.2±0.3	4.1±0.1	4.1±0.3
**Total protein (g/dL)**	5.9±0.4	6.5±0.5*	5.9±0.3	6.6±0.7*
**CPK (IU/L)**	686±264	958±700	436±197	703±365*
**Creatinine (mg/dL)**	1.21±0.2	1.24±0.3	1.31±0.1	1.28±0.2
**Urea (mg/dL)**	30.8±3.5	24.4±3.6*	30.9±3.5	25.5±3.9*

S1 Table. Plasma biochemistry. Control (n=10) and MetS (n=11). *p<0.05 *vs.* control.
