# Supplementary material for: Development and characterization of an experimental model of diet-induced metabolic syndrome in rabbit
Source: PLoS One. 2017 May 23;12(5):e0178315. doi: 10.1371/journal.pone.0178315 (PMC5441642; doi:10.1371/journal.pone.0178315)
Supplement: S2 Table — (PDF) [file pone.0178315.s002.pdf]

	Week 14		Week 28	
	**Control**	**MetS**	**Control**	**MetS**
**Cholesterol**	3.8±0.1	3.8±0.1	3.8±0.1	3.9±0.1
**FA-CH_3**	4.9±0.3	4.8±0.3	4.9±0.4	5.1±0.6
**FA=CH-CH_2-CH=**	1.5±0.2	1.5±0.2	1.4±0.1	1.5±0.2
**Glycerol**	1.5±0.1	1.8±0.6	1.4±0.3	1.5±0.4
**O-Acetylcarnitine**	0.52±0.07	0.58±0.11	0.51±0.16	0.46±0.13
**2-Hydroxy 3-Methylacetate**	4.8±0.5	4.7±0.8	4.8±0.6	4.5±0.5
**HDL3 apolipoproteins**	2.5±0.1	2.3±0.2	2.5±0.1	2.4±0.1

S2 Table. Metabolomic analysis of fatty acids and related compounds. Control (n=10) and MetS (n=11).
