# Supplementary material for: Development and characterization of an experimental model of diet-induced metabolic syndrome in rabbit
Source: PLoS One. 2017 May 23;12(5):e0178315. doi: 10.1371/journal.pone.0178315 (PMC5441642; doi:10.1371/journal.pone.0178315)
Supplement: S3 Table — (PDF) [file pone.0178315.s003.pdf]

	Week 14		Week 28	
	**Control**	**MetS**	**Control**	**MetS**
**Glutamate**	2.6±0.4	2.9±0.9	3.1±0.6	2.8±0.5
**Glutamine**	4.9±0.5	5.3±1.5	3.9±0.8	4.7±1.3
**Glycine**	0.7±0.1	1.0±0.2*	0.7±0.1	0.8±0.1
**Histidine**	1.7±0.4	1.4±0.3	1.4±0.2	1.6±0.4
**Isoleucine**	4.9±0.3	5.5±0.9	5.1±0.7	4.8±0.9
**Leucine**	5.9±0.7	6.2±0.9	5.9±0.7	5.7±1
**Lysine**	5.0±0.4	5.5±0.9	4.9±0.6	4.8±0.9
**Methionine**	0.7±0.2	0.7±0.3	0.9±0.3	0.7±0.2
**N-Acetyltyrosine**	1.7±0.4	1.5±0.2	1.6±0.2	1.5±0.3
**Phenylalanine**	1.4±0.1	1.5±0.3	1.4±0.3	1.4±0.4
**Proline**	3.2±0.5	3.5±0.9	3.8±0.8	3.6±0.9
**Sarcosine**	0.31±0.07	0.36±0.09	0.29±0.08	0.31±0.09
**Valine**	4.6±0.4	4.9±0.9	4.3±0.7	4.6±0.8

S3 Table. Metabolomic analysis of amino acids. Control (n=10) and MetS (n=11).
