# Supplementary material for: Development and characterization of an experimental model of diet-induced metabolic syndrome in rabbit
Source: PLoS One. 2017 May 23;12(5):e0178315. doi: 10.1371/journal.pone.0178315 (PMC5441642; doi:10.1371/journal.pone.0178315)
Supplement: S4 Table — (PDF) [file pone.0178315.s004.pdf]

	Week 14		Week 28	
	**Control**	**MetS**	**Control**	**MetS**
**2-Hydroxy isobutyrate**	0.5±0.1	0.7±0.2	0.5±0.1	0.6±0.2
**2-Metylglutarate**	3.3±0.5	3.1±0.4	3.4±0.8	3.1±0.6
**3-Aminoisobutyrate**	1.7±0.1	1.9±0.3	1.8±0.1	1.9±0.4
**3-Hydroxybutyrate**	2.4±0.3	2.4±0.2	2.3±0.3	2.7±0.6
**Acetate**	0.5±0.1	0.5±0.1	0.5±0.1	0.5±0.1
**Cellobiose**	4.7±0.8	5.1±1.7	5.9±1.6$^{\$}$	6.3±1.6$^{\$}$
**Choline**	0.69±0.15	0.79±0.13	0.71±0.2	0.63±0.11
**Citrate**	1.1±1.0	1.4±0.2	1.2±0.1	1.4±0.5
**Creatine**	0.49±0.03	0.50±0.11	0.50±0.08	0.48±0.11
**CK**	0.49±0.04	0.49±0.07	0.49±0.07	0.47±0.08
**Creatinine**	0.84±0.03	0.91±0.14	0.89±0.12	0.83±0.15
**Dimethylamine**	0.17±0.02	0.18±0.03	0.16±0.19	0.16±0.04
**Ethanol**	1.13±0.10	1.29±0.30	1.43±0.14	1.3±0.29
**Formate**	0.15±0.04	0.14±0.04	0.14±0.02	0.13±0.04
**Fumarate**	0.05±0.01	0.05±0.01	0.05±0.01	0.04±0.01
**Glycerate**	2.4±0.5	2.1±0.3	1.9±0.5$^{\$}$	1.9±0.5$^{\$}$
**Isobutyrate**	0.81±0.01	0.83±0.04	0.9±0.10	0.8±0.06
**Kynurenine**	1.9±0.1	2.1±0.4	1.9±0.2	1.9±0.4
**Myoinositol**	1.8±0.2	1.8±0.3	2.1±0.2	2.1±0.5
**N-acetylcisteine**	1.7±0.2	1.7±0.3	1.7±0.1	1.7±0.2
**Succinate**	0.17±0.02	0.19±0.06	0.19±0.04	0.17±0.04
**Succinylacetone**	1.1±0.1	1.4±0.4	1.2±0.2	1.15±0.3
**Trimethylamine**	0.22±0.01	0.24±0.04	0.23±0.03	0.21±0.04
**Trimethylamine-N-oxide**	0.53±0.29	0.61±0.23	0.46±0.12	0.97±0.9
**Urea**	0.19±0.09	0.14±0.08	0.18±0.03	0.21±0.08

S4 Table. Metabolomic analysis of other metabolites. Control (n=10) and MetS (n=11). $^{\$}p<0.05$ vs. week 14.
